# Cutaneous changes in diabetic patients: Primed for aberrant healing?

**DOI:** 10.1111/wrr.13108

**Published:** 2023-07-19

**Authors:** Vivien Y. Chen, Lindsey G. Siegfried, Marjana Tomic-Canic, Rivka C. Stone, Irena Pastar

**Affiliations:** Wound Healing and Regenerative Medicine Research Program, Dr. Phillip Frost Department of Dermatology and Cutaneous Surgery, University of Miami Miller School of Medicine, Miami, Florida, USA

**Keywords:** diabetes, diabetic foot ulcer, glycation, hyperglycaemia, inflammation, neuropathy, skin, wound healing

## Abstract

Cutaneous manifestations affect most patients with diabetes mellitus, clinically presenting with numerous dermatologic diseases from xerosis to diabetic foot ulcers (DFUs). Skin conditions not only impose a significantly impaired quality of life on individuals with diabetes but also predispose patients to further complications. Knowledge of cutaneous biology and the wound healing process under diabetic conditions is largely limited to animal models, and studies focusing on biology of the human condition of DFUs remain limited. In this review, we discuss the critical molecular, cellular, and structural changes to the skin in the hyperglycaemic and insulin-resistant environment of diabetes with a focus specifically on human-derived data. Elucidating the breadth of the cutaneous manifestations coupled with effective diabetes management is important for improving patient quality of life and averting future complications including wound healing disorders.

## 1 | INTRODUCTION

Cutaneous manifestations impact an estimated 70% of diabetic patients worldwide.^1^ Common clinical findings include xerosis and cutaneous infections, which can predispose patients to additional complications.^1^ Development of chronic foot ulcers is one of the most severe complications of diabetes, resulting in increased risk for limb amputations and a significant reduction in overall quality of life.^2,3^ Diabetes mellitus has also been associated with numerous dermatologic diseases including acanthosis nigricans, acrochordons, diabetic dermopathy, granuloma annulare, necrobiosis lipoidica, scleredema diabeticorum, and bullosis diabeticorum.^4^

These diseases arise from the pathologic hyperglycaemia and insulin resistance characteristic of diabetes mellitus. Hyperglycaemia has been shown to inhibit keratinocyte proliferation and migration, alter protein biosynthesis, induce endothelial cell apoptosis, and impair phagocytosis and chemotaxis of immune cells.^5–11^ Formation and accumulation of advanced glycation end products (AGE) in the skin can also disrupt collagen function and immune cell activity, potentially delaying wound healing and increasing susceptibility to infection.^12–14^

Proper blood glucose control, along with awareness and management of early cutaneous signs such as skin moisturization and orthotic devices, can avert further skin complications in diabetic patients. Diabetic foot ulcers (DFUs) are hallmarks of advanced disease and are frequently generalised to represent cutaneous wound healing behaviour in diabetes. However, evidence supporting impaired or delayed healing of *acute* wounds in the diabetic population, for example, surgical wounds, is lacking. In fact, much of the current understanding of structure and molecular composition of diabetic skin has been obtained from various diabetic animal models.^15,16^ Elucidating the breadth of subclinical and clinically apparent cutaneous effects of diabetes on skin biology is of importance and may contribute to novel treatment strategies aiming to prevent further complications, including impaired wound healing. This review will discuss the structural and molecular changes in non-injured diabetic skin in patients with type 1 (T1D) and type 2 diabetes (T2D) that lead to early cutaneous manifestations, with a focus on human-derived studies.

## 2 | EPIDERMAL BARRIER CHANGES IMPACTED BY DIABETES

**FIGURE 1:**
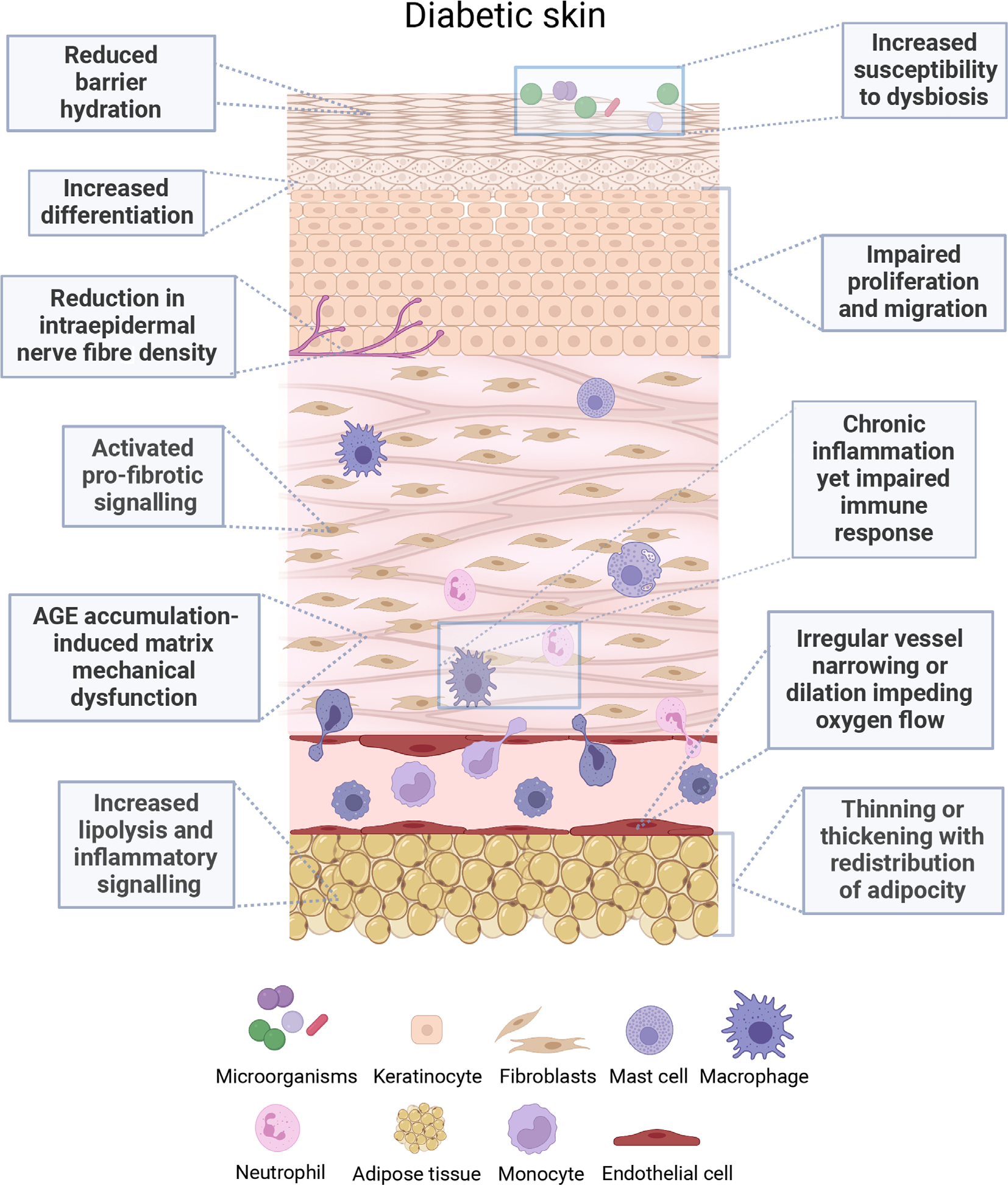
Representation of diabetes-induced cutaneous changes affecting epidermis, dermis, peripheral nerves, immune cells and subcutaneous tissue. AGE, advanced glycation end products.

Patients with diabetes often suffer from dry skin, presenting clinically with irritation, itch, and increased fragility. Skin breakdown further facilitates the route of entry for bacterial or fungal microorganisms and increases the risk of wound formation (Figure 1).^7,17^ Xerosis develops through several mechanisms related to pathologic hyperglycaemia. Non-enzymatic glycation of collagen fibres increases crosslinking and modifies sidechains to interfere with cell–collagen interactions, causing decreased flexibility and solubility (Table 1).^12^ Consequently, patients with diabetes have impaired skin elasticity compared with healthy controls.^18,19^ Structural changes may also be reflected on the gene level, as a whole genome expression analysis of lower leg skin in diabetic patients found downregulated genes in cell adhesion molecule pathways (NCAM1 and L1CAM) and the collagen family members such as type IX collagen and Procollagen C-Endopeptidase Enhancer 2.^20^ Interestingly, of 56,318 genes expressed in skin, only 182 are found to be significantly differentially expressed between diabetic and non-diabetic skin. Similarly, transcriptomic analyses of tissue samples collected from non-ulcerated and non-neuropathic diabetic foot skin showed minimal differences in expression levels of mRNA and post-transcriptional regulators microRNA (miRNA) when compared with location matched healthy foot skin controls.^5^ Although intact diabetic foot skin shows small differences from non-diabetic foot skin at transcriptional level, its response to challenges (such as wounding, infection, or neuropathy) may be very different.

The impact of diabetes on stratum corneum (SC) function, specifically SC hydration and trans-epidermal water loss (TEWL), reveals conflicting findings.^19,21^ SC hydration is primarily regulated by a mix of natural moisturising factors (NMFs) that attract and bind water molecules, as well as the physical barrier created by corneocytes and intercellular lamellar lipids such as ceramides, cholesterol, and free fatty acids. The hygroscopic molecules primarily include filaggrin-derived amino acids, lactate, and urea.^22,23^ Clinically, some studies have observed decreased SC hydration and TEWL in patients with diabetes, associated with poor levels of glycaemic control and older patient age.^21,24^ These findings correlate with murine models that have demonstrated reduced skin hydration through decrease in hyaluronic acid, decreased epidermal lipid synthesis, and lamellar body number in association with increased serum AGE.^24,25^ Another clinical study in diabetic patients controlling for age and sex did not observe differences in hydration and TEWL.^19^ Factors potentially contributing to the contradictory findings are patient age, use of lipid-lowering medications, and presence of obesity between different study populations. Further studies controlling for such confounding factors may better elucidate the impact of diabetes on the SC properties.

One of the greatest areas of concern with skin fragility is the foot, where DFUs form in the context of advanced diabetes and peripheral neuropathy. Xerotic foot skin in patients with diabetes demonstrated greater stiffness, thinning, and decreased hydration than in non-diabetic patients, without affecting TEWL.^7^ Clinically, this correlated with an up to three-fold increased number of superficial fissures, as well as severe scaling. Notably, specific markers such as ceramide concentration, amino acids, serine, and total proteins were found elevated in diabetic xerotic foot skin compared with non-diabetic xerotic foot skin.^7^ The difference could reflect the primary mechanism of dry skin in diabetes arising from additional cofounders such as collagen disruption, rather than simply a decline of NMFs or inter-cellular lipids demonstrated in other conditions such as senile xerosis. Nevertheless, moisturising can improve xerotic changes and multiple randomised controlled trials on foot xerosis in diabetes have demonstrated the enhanced efficacy of moisturisers supplemented with natural moisturising factors such as urea, lactic acid, and amino acids.^26–28^ Recent evidence also suggests foot hydration levels could be a non-invasive indicator for foot sole deterioration. Using a Terahertz radiation, pulsed laser-based imaging approach, patients with diabetes demonstrated decreased water content in foot skin, particularly in the heel centre, and lower levels further correlated with degree of diabetic peripheral neuropathy.^29^ Methods to measure foot skin hydration could be future diagnostic tool to predict neurological deterioration and therefore risk of developing DFU.

### 2.1 | Effects of diabetes on keratinocytes

Epidermal keratinocytes serve not only as important components of the physical and chemical barrier of the skin but play an active role in cell signalling and cutaneous immune function.^30–33^ Cutaneous disorders like xerosis underscore the systemic changes caused by diabetes as well as the multifunctional activity of keratinocytes in reflecting diabetic pathology.

Keratinocyte activation resulting in proliferation and migration are key components of the re-epithelialisation process following barrier breach.^30–32^ During skin repair, keratinocyte motility is achieved by shifting keratin (K) expression to K6, K16, and K17, facilitating increased cellular flexibility to migrate.^34^ Motility is also supported by overall loss of cell-to-cell adhesions and matrix-metalloproteinase (MMP) proteolysis of extracellular matrix (ECM) proteins.^35^ The hyperglycaemic environment of diabetes inhibits keratinocyte migration by reduced MMP activity in the context of increased tissue inhibitor of metalloproteinase (TIMP) and TGF-β secretion.^6^ Keratinocytes can further exacerbate hyperglycaemia by autoregulating glucose transporters and thereby uptake.^36^ Although hyperglycaemic conditions of cell culture in vitro and diabetic murine models in vivo demonstrate altered keratinocyte morphology and number,^5,37^ histological evaluation of human diabetic foot skin and normal foot skin reveals no observable differences in epidermal morphology, dermal collagen orientation, fibres composition, skin thickness, or leptin receptor signal, with the upregulation of S100 calcium binding protein A9 (S100A9) detected in some (33%) but not all diabetic foot skin samples.^5,36,38^ Conversely, hyperglycaemia, not hyperinsulinemia, was found to be associated with suppressed keratinocyte proliferation in T1D mouse model in vivo,^38^ suggesting limited translation to the human condition.

Despite these functional and morphological changes to keratinocytes in diabetic conditions, some skin pathologies associated with diabetes exhibit opposite, hyper-proliferative effects. Acanthosis nigricans, an associated skin disorder causing hyper-pigmented, thickened plaques in intertriginous body areas, arises from hyperinsulinemia stimulating insulin-like growth factor, with other potential mediators such as epidermal and fibroblast growth factor receptors stimulating keratinocyte and fibroblast proliferation.^39^ DFUs are characterised by a hyper-proliferative but non-migratory epidermis, with several factors contributing to keratinocyte dysfunction. Altered signalling pathways including activation of Wnt-pathway, overexpressed c-myc and suppressed TGF-β promote keratinocyte mitotic division beyond the basal layer, with modulated expression of keratinocyte differentiation markers such as K1/K10, filaggrin, involucrin, and transglutaminase 1.^40,41^ Epigenetic changes including miRNA deregulation and histone modifications also inhibit keratinocyte migration in DFU.^30,42–46^

In keratinocytes, hyperglycaemic conditions cause mitochondrial reactive oxygen species (ROS) overproduction and a decrease in endogenous antioxidant production in a dose-dependent manner, leading to oxidative damage, accelerated keratinocyte apoptosis, and mitochondrial DNA damage (Table 1).^47,48^ ROS such as nitric oxide has potent regulatory effects on keratinocyte proliferation and differentiation, but the point at which ROS or other signalling molecules transition from functional and necessary to destructively dysfunctional has not yet been elucidated.^49,50^ Hyperglycaemic conditions in keratinocytes also increase sensitivity to ultraviolet exposure, with an increased release of exosomes and their miRNA content that may influence inter-cellular communication.^51^

## 3 | DIABETES ALTERS IMMUNE RESPONSE

Diabetes is classically associated with a state of chronic low-grade inflammation that significantly impairs the immune system, impacting infection resolution and wound healing. Diabetes has been shown to modify the bone marrow architecture and function, lowering the number of sympathetic nerve terminal endings and mobilising immature endothelial progenitor cells, monocytes, and macrophages into the bloodstream, contributing to inflammatory and angiogenic dysregulation.^52–54^ Regardless of neuropathy, cutaneous presence of immune cells is elevated in diabetic patients, and the impaired migratory characteristics of the present immune cells are indicative of overarching immune regulatory dysfunction (Figure 1 and Table 2).^55,56^ Recruitment and function of distinct immune cell types into the skin are also modified to contribute to dysregulated inflammation as outlined for macrophages, monocytes, mast cells, and neutrophils.

### 3.1 | Macrophages

Both tissue-resident and bone marrow-derived macrophages in diabetic patients have an impaired phenotype transition from M1 to M2 like, favouring an M1 pro-inflammatory phenotype that contributes to the persistent inflammatory state (Table 2).^9,57,58^ In vitro mechanistic studies have demonstrated that high glucose primes macrophages for increased expression of pro-inflammatory cytokines (tumor necrosis factor-α [TNF-α], interleukin-1 [IL-1], IL-6), further exacerbated with macrophage activation or with a hypoxic environment.^59,60^ Epigenetic modifications also sustain the pro-inflammatory disposition.^46^ Patients with T2D have serum peripheral monocytes with elevated levels of histone methyltransferase MLL1, which upregulates pro-inflammatory cytokine IL-6.^61,62^ T2D patients display elevated cell-free mtDNA serum levels as well, which induces AIM2 inflammasome-mediated inflammation, and IL-1β and IL-18 secretion in macrophages.^63,64^ Macrophages also demonstrate decreased phagocytic ability in diabetic patients, contributing to impaired host defence. A hyperglycaemic environment suppresses genes involved in mediating bacterial phagocytosis, and efferocytosis, the phagocytic clearance of apoptotic cells.^59^ In diabetic murine models, efferocytosis by macrophages is impaired.^65^ Monocytes and macrophages from diabetic patients also demonstrate impaired phagocytic activity in correlation to glycaemic control (Table 2).^9,10^

The deregulated polarisation and function of macrophages have also been studied in the context of impaired wound healing in diabetes.^66–70^ Interestingly, several recent studies have shown that decreased macrophage recruitment contributes to DFUs, suggesting that inflammatory response does not recapitulate the response typically detected in acute wound healing or in murine models of diabetes.^71^ This was further confirmed by single-cell transcriptomic analyses of DFUs that showed increase of M1 macrophages in healing DFUs, suggesting that activation of acute-like inflammatory response supports the progression of healing.^72^ From investigating hyperglycaemic effects on macrophage dysfunction, pre-clinical studies have demonstrated prospective therapeutic targets to rescue diabetes-induced impairment in macrophage function and improve wound healing in animal models.^73–76^

### 3.2 | Monocytes

Monocytes recruited to the tissue play key roles during inflammation and pathogen challenge, as monocyte-derived macrophages directing inflammatory response and as antigen-presenting cells.^77^ Alteration in sympathetic innervation and activation in the bone marrow are known to impact the haematopoietic stem cell niche and disrupts stem cell mobilisation and differentiation.^53,78,79^ Murine models have established that low-grade chronic inflammation in diabetes induces myeloid bias in haematopoietic stem and progenitor cells, increasing monocyte production in the bone marrow.^80^ Diabetic patients also reflect elevated levels of circulating inflammatory monocytes (CD14+ CD16−).^79,81^ Chemotaxis and activation of monocytes in diabetes are also modified, as hyperglycaemia contributes to resistance of vascular endothelial growth factor-induced chemotaxis (Table 2).^11,82–84^ A genomic assay of forearm skin biopsies in diabetic patients revealed an impaired migratory profile of immune cells, including dysregulated migration of monocytes, dendritic cells, and antigen-presenting cells.^56^ Elevated glucose also promotes NADPH-oxidase mediated toll-like receptor expression and activity in monocyte activation, as well as increased pro-inflammatory cytokine production, contributing to increased inflammatory responses.^85,86^

### 3.3 | Mast cells

Mast cells participate in several processes of inflammation, neovascularisation, and wound healing via degranulation, release of mediators, and recruitment and interaction with other cells including macrophages, neutrophils, endothelial cells, and fibroblasts.^58^ Mast cell activation response to stimuli vary based on tissue location and age, and shift from regenerative to tissue repair mechanism during mammalian development is associated with maturation and activation of mast cells.^87^ Their role in scarring is well established, and priming skin prior to surgery with mast cell inhibitor epigallocatechin-3-gallate decreases mast cells and contributes to significant post-surgical scar reduction.^87–89^

In diabetes, mast cells play a role in disease progression. T1D patients demonstrated increased mast cells infiltration in the pancreatic islets with induced β-cell death.^90^ Mast cells also help progress lipid retention, vascular remodelling, and atherosclerosis in T2D through promoting adipose tissue inflammation, although activity depends on the adipose anatomical location.^90^ Unwounded skin in diabetic patients revealed increased numbers of degranulated mast cells associated with inflammatory infiltrates in the dermis (Table 2).^56,58^ While the absence of mast cells impairs early skin wound healing in mice, non-degranulated mast cells in skin are essential for proper adult wound healing.^58,90^ Therefore, therapies towards mast cell stabilisation and regulation of degranulation have been proposed to improve the wound healing capacity of diabetic skin.^58,90^

### 3.4 | Neutrophils

Neutrophils play a vital role in first line defence during early inflammatory response, clearing foreign debris and bacteria upon barrier breach and coordinating immune response. Neutrophils in diabetic patients exhibit increased release of pro-inflammatory cytokines (TNF-α, IL-8, IL-1β), dysregulated ROS generation, and inflammatory cell death with increased release of neutrophil extracellular traps (NETosis) measured by increased marker citrullinated histone H3 (Table 2).^86,91,92^ Increased NETosis continues to be present in DFUs, along with decreased neutrophil number at wound edge and deregulation of transcriptional networks involving FOXM1, STAT3, and TREM1 that support neutrophils and other immune cells.^71,93^ The diabetic environment also alters neutrophil function, contributing to a weakened response against infection.^94^

## 4 | DERMAL CHANGES DUE TO DIABETES

### 4.1 | Fibroblasts

Fibroblasts and their different phenotypes and lineages are vitally involved in physical cutaneous structure, skin homeostasis, and wound healing processes of inflammation, angiogenesis, and granulation tissue formation.^95–100^ Although advances of high-resolution -omic technologies revealed complexity and plethora of fibroblast phenotypes and their function, little is known about how diabetes affects these distinct populations. Hyperglycaemia exacerbates the inhibitory effect of ischemia on myofibroblast differentiation.^101^ Fibroblasts incubated in hyperglycaemic conditions exhibit senescence and accelerated apoptosis.^102^ Recent single-cell transcriptomic identified a unique population of fibroblasts associated with healing of DFUs.^72^

Emerging evidence points to the involvement of miRNAs in the complex wound microenvironment and a potential major point of differentiation between diabetic and normal wound healing.^5,103^ miRNA profiling of primary dermal fibroblasts from diabetic and healthy human foot skin identified subtle changes in the regulation of miRNAs between diabetic and healthy foot skin.^5^ Fibroblasts isolated from diabetic wounds express miR-27–3p, a miRNA associated with promoting insulin resistance and diabetic retinopathy, at significantly higher levels than fibroblasts isolated from normal wounds.^104^ miR-27–3p suppresses fibroblast proliferation and migration, increases apoptosis in vitro, and delays wound healing.^104^ Furthermore, paired mRNA–miRNA transcriptomic analyses of human fibroblasts derived from DFUs revealed reveals impairments in cellular functions such as cell migration, proliferation, activation of cell differentiation, and senescence (Table 1),^103^ features that can be re-programmed using induced pluripotent stem cells approach from a non-healing diabetic to pro-regenerative fetal-like healing phenotype.^103,105,106^

Diabetes is also known to increase the amount of AGE (Table 1 and Figure 1), present in many tissues including skin.^107^ Glucose, among other reducing sugars, reacts with amino groups on proteins, lipids, and nucleic acids via the Maillard reaction, eventually leading to the accumulation of AGE.^108^ AGE receptors (RAGE) and post-receptor signalling pathways have been identified, and mechanisms involving RAGE have been implicated in the tissue changes and damage consequential to diabetes.^109^ AGE-derived oxidant stress, tissue-specific inflammation, growth, and apoptosis enhanced by the hyperglycaemic environment have been shown to contribute to pathogenesis of long-term diabetic complications.^110^ Prominent AGE signal was found in the dermis of diabetic skin combined with an increase in RAGE -positive fibroblasts compared with healthy non-diabetic skin.^110^ Additionally, AGE levels in diabetic skin are higher in individuals with distal sensorimotor polyneuropathy (DSP) and are associated with DSP severity.^111^ In vitro treatment of human fibroblasts with glyoxal, a glycation reaction product, revealed a decrease in fibroblast proliferation, increased tensile strength, increased adherence to ECM, downregulated focal adhesion kinase and exhibited failure to extend filipodia, demonstrating multiple points of hindrance to fibroblast migration and proliferation as a result of AGE treatment.^112^ Treatment of fibroblasts with rosiglitazone, a peroxisome proliferator-activated receptor-gamma ligand, attenuated the effects of AGE in vitro.^113^ Connective tissue growth factor (CTGF), a key player in fibroblast-ECM production and angiogenic factor, has been identified as a potential mediator between increased AGE and tissue fibrosis in diabetes.^114^ Human skin fibroblasts cultured in the presence of AGE show upregulation of CTGF compared with control fibroblasts.^114^ A separate study showed that apoptosis is induced in human fibroblasts cultured with the most abundant type of AGE, known as *N*^ϵ^-(carboxymethyl)lysine.^115^

### 4.2 | Extracellular matrix

Fibrosis of the heart, kidney, and liver are well-established forms of diabetes-related end-organ damage, and diabetic skin also responds to systemic pro-fibrotic signals.^116–118^ Hyperglycaemia, lipotoxic injury, and insulin resistance promote fibrosis through the stimulation of fibroblasts, immune cells, and vascular cells, and may activate a fibroblast-like phenotype in epithelial and endothelial cells.^119^ The MMP/TIMP ratio is also unbalanced in diabetic skin, potentially due to chronic pro-inflammatory and fibrotic signals released in the presence of high glucose (Table 1).^120^

Skin autofluorescence, which correlates with advanced glycation, is significantly higher in individuals with both T1D and T2D compared with age-matched non-diabetic patients.^121^ In individuals with T1D, autofluorescence was also found to predict development of DFU and limb amputations across 10 years of observation.^122^ Pentosidine amount and 370/440 nm fluorescence intensity in skin collagen as parameters of AGE accumulation are also tightly correlated with diabetes duration and have demonstrated capacity as predictors of long-term complications like retinopathy and elevated creatinine.^123^ Advanced glycation is a long-term process thus predominantly affecting long-living proteins like collagen and other ECM structural components.^108^ Incubation of fibroblasts with AGE-bovine serum albumin revealed upregulations in RAGE, TGF-β1, collagen I, and collagen III as well as MMP-2 activation.^124^ Chronic stimulation of fibroblasts demonstrated that collagen I synthesis is a result of RAGE upregulation, whereas collagen III synthesis is a result of both TGF-β1 and RAGE upregulation.^124^ The tensile strength of diabetic skin, measured by average maximum stress and average modulus, is significantly lower than normal skin and can be attributed to the biomechanical effects of glycation.^125^

In addition to endogenous AGE accumulated via diabetes-related metabolic damage, AGE content in skin is also correlated with lifestyle factors such as diet, smoking, and sun exposure.^126^ The development and progression of T2D specifically are associated with dietary factors, and the potential benefit of lifestyle modifications in preventing major diabetes complications may also benefit the skin in attenuating AGE accumulation.^127^

## 5 | EFFECTS OF DIABETES ON SUBCUTANEOUS ADIPOSE TISSUE

The involvement of adipose tissue systemically in diabetes is extensive and reviewed elsewhere^128–130^ clearly demonstrating that diabetes impacts both visceral and subcutaneous adipose fat tissue (Figure 1). Adipose tissue that accumulates around abdominal viscera and intra-abdominal solid organs contributes to metabolic obesity and is functionally distinct from subcutaneous adipose tissue. Visceral adipose tissue demonstrates increased lipolytic activity and production of pro-inflammatory cytokines, and links to insulin resistance and cardiovascular risk.^131–135^ Diabetes medications such as the thiazolidinedione family may induce fat redistribution as a mechanism of action in their treatment of diabetes.^136^ The distribution ratio of visceral to subcutaneous fat is also affected by sex and race/ethnicity.^137^

There is conflicting evidence regarding diabetes-induced changes to the subcutaneous adipose tissue, which may point to redistribution and body site-specific changes in diabetes rather than a universal thinning or thickening of adipose tissue. Studies demonstrate a lack of association between increased subcutaneous fat and insulin resistance but conflict over whether these fat deposits are ‘less pathogenic’ than visceral fat or have actual protective benefits against metabolic morbidity.^131,132,135,138–140^ Studies have found diabetic patients exhibit thinned skin and thinned subcutaneous layers such as on the foot, back, and hand, whereas other studies using the same method of measurement found that slightly thicker subcutaneous layers on the foot and back, as well as the anterior abdominal wall.^141–143^ Additional evidence supports thinned subcutaneous layers independent of sex, race/ethnicity, and visceral-subcutaneous ratio in diabetic patients.^144^ Conflicting observations of subcutaneous adipose tissue thickness warrants further investigation into the factors involved such as demographics and comorbidities.

Diabetic patients demonstrate key characteristics of adipose tissue dysfunction within subcutaneous fat including enlarged adipocytes, increased inflammatory markers such as M1/M2 macrophage ratio, TNF-α secretion, as well as increased lipolysis and deregulation of adipogenesis and morphology.^37,145,146^ Similarly, healthy patients genetically predisposed to T2D demonstrate adipocyte hypertrophy independent of body mass index, associated with increased Wnt signalling activity indicating impaired adipocyte differentiation. They also reveal increased markers of inflammation (IL1-β, IL10, TNF-α) and macrophage infiltration, and early signs of adipose tissue remodelling and fibrosis, suggesting adipose tissue dysfunction may predate disease onset. Communication between adipocytes and macrophages as well as other inflammatory cells directly contributes to cutaneous wound healing.^147–149^ Furthermore, adipocyte-derived cells at the wound edge can also differentiate into myofibroblasts, the dominant ECM-producing cell type during the proliferative stage of wound healing.^149^

Potential advancements in diabetes treatment options include replenishing healthy adipose tissue function, either via administration of specific hypoglycaemic, anti-inflammatory adipokines or adipose tissue transplantation, which have demonstrated promising efficacy in rodent models but have not yet been replicated clinically.^150–154^ Human-derived adipose stem cells have also been identified as a promising therapeutic for enhancing diabetic wound healing.^155–157^ While changes in distribution of subcutaneous layers remain unclear, diabetic patients experience adipose tissue redistribution and dysfunction that promote insulin resistance, fat storage, and deregulated inflammation and remodelling processes. Further advancements towards reversal, replacement, or supplementation of healthy adipocyte function may prove promising in attenuating adipocyte-driven pathology in diabetic complications including cutaneous wound healing.

## 6 | INFECTION RISK AND CUTANEOUS MICROBIOME IN DIABETIC PATIENTS

Resulting from the low-grade chronic inflammatory state, diabetes promotes inappropriate immune function that places patients at increased risk of infection. A dysfunctional skin barrier from glycaemic changes to collagen and SC may also present easier entry of pathogens for infection. Diabetic patients report about a two-fold increased risk of skin and soft tissue infections, most commonly including cellulitis and infected ulcers, with decreased resolution rates and increased complications associated with poor glycaemic control.^158,159^ Certain antimicrobial peptides such as RNAse7 are downregulated in the skin of diabetic patients regardless of ulcer presence,^160^ decreasing host defence against infection.

Evidence indicates that the skin microbiome composition is altered in diabetic patients (Figure 1). A case-control study of cutaneous foot microbiome in T2D patients demonstrated increased abundance of *Staphylococcus aureus* and decrease in commensal *Staphylococcus epidermidis* compared with healthy skin.^161^ Increased abundance of *S. aureus* may arise from the epidermal barrier defects and hyperglycaemia, which increases glucose nutrient availability for *S. aureus* to enhance expression of virulence factors.^162,163^ This is in line with the complex microbiome characterised with antibiotic-resistant *S. aureus* and anaerobic bacteria associated with the non-healing outcomes in DFU.^164^ Another study of foot skin microbiome found that diabetic patients had a different microbiome composition, with increased *Trichophyton rubrum* and decreased fungal diversity.^165^ *T. rubrum* is a common dermatophyte contributing to onychomycosis and tinea pedis, reflecting the increased prevalence of fungal manifestations among the diabetic patient population.^166,167^

## 7 | MICROVASCULATURE CHANGES AND PERIPHERAL NEUROPATHY DUE TO DIABETES

Hyperglycaemia is believed to cause microvascular disease in diabetes, with synergistic contribution from hypertension, dyslipidaemia, smoking, and duration of diabetes.^168^ Not limited to diabetes, microvascular and macrovascular dysfunction are independently associated with prediabetes and hyperglycaemia as well.^169^ Free fatty acid-derived endothelial injury causes insulin resistance and inflammation in insulin target tissues, resulting in inhibition of nitric oxide-mediated vasodilation.^8^ Vascular response to heat stress is also impaired in diabetes compared with both young and old non-diabetic individuals, indicating that the slowed, less prominent response may contribute to poor wound healing of burns in diabetic individuals.^141,170^

Microangiopathy, eventually leading to retinopathy, nephropathy, and peripheral neuropathy in diabetes, is the result of the vascular lumen becoming unevenly narrowed or dilated due to the excess deposition of type IV collagen and laminin, the thickening of basement membranes, and the accumulation of endothelial cells.^171–173^ The cumulative effects of macroangiopathy due to atherosclerosis, vasculitis and microangiopathy contribute to skin atrophy and nail abnormalities due to chronic lack of oxygen.^173^ Prostacyclin I2 analogue increases blood flow in the foot skin of diabetic patients in correlation with reducing plasma thrombomodulin levels, an endothelial cell surface receptor for thrombin important in protein C-regulated coagulation.^174^ Peripheral hypercoagulation contributing to inadequate tissue perfusion may also promote the dysregulated wound healing cascade observed in DFUs.

Cutaneous microangiopathy in patients with diabetes also precludes future deterioration of blood glucose tolerance and severity of peripheral neuropathy.^175–177^ Diabetic peripheral neuropathy is estimated to affect 30% of diabetic patients, a greater proportion of those with T2D.^178^ Multiple studies have shown reductions in intraepidermal nerve fibres density in skin biopsies from diabetic patients.^179,180^ As a diabetes complication associated with increased cardiovascular mortality and severe systemic manifestations, peripheral neuropathy also causes dry skin, loss of sweating, and loss of skin barrier function.^181^ These cutaneous changes can contribute to the development of skin fissures, producing susceptibility to microorganisms in the context of an already deregulated immune system.^181^ Some studies suggest altered leukocyte composition in association with development of diabetic peripheral neuropathy, with increased neutrophil-to-lymphocyte ratio and increased basophil and CD4+ T-cell populations.^182,183^ Diabetic patients demonstrate significantly decreased periglandular nerve terminals,^184^ and various neuropathy screening methods have found asymmetry of sensorimotor neuropathy between extremities to correlate with disease severity and DFUs.^185,186^

## 8 | PHYSIOLOGICAL ACUTE WOUND HEALING IN DIABETIC INDIVIDUALS

The pathogenesis and pathophysiology of DFUs is an active area of research, but comparatively little is documented regarding the healing behaviour of acute wounds in diabetic patients.^45,187–189^ Frequent reference is made to impaired post-operative wound healing in diabetic patients, for example, poor healing of post-surgical wounds, yet clinical evidence supporting this assertion is scant. Interestingly, surgical site infection was the only outcome found to be significantly increased post-operatively in diabetic patients as compared with non-diabetic individuals.^190,191^ To this end, meta-analysis revealed a significant association between diabetes and incidence of surgical site infection across multiple types of surgeries independent from body mass index, and this association was maintained independent from perioperative hyperglycaemia.^192^ Even this association has not been consistently reproduced; for example, no association between diabetes and post-operative infection was found on analysis of infection risk factors in patients undergoing bariatric surgery.^193^ Notably, studies have not supported a difference in post-surgical clinical healing outcomes—including delayed wound closure and abnormal scarring—in diabetic and non-diabetic wounds.^190,191^ For example, while AGE accumulation in diabetic skin impeding normal dermal function wound be expected to affect wound healing and the mechanical properties of scars, there are few reports to support that significantly impacts clinical wound healing outcomes in patients.^14^ Comorbidities, indications for surgery, and types of surgery impact not only the nature of the wound but also the systemic environment in which the wound is required to heal. Future studies are needed to observe the post-operative healing of diabetic and non-diabetic individuals to document patterns in healing rates, complication risks, and abnormal scarring.

## 9 | CONCLUSIONS AND CLOSING REMARKS

Diabetes causes significant structural and molecular changes to the skin, priming for development of cutaneous complications such as DFU (Figure 1). Pathologic uncontrolled hyperglycaemia contributes to impaired epidermal barrier and skin fragility. Altered keratinocyte and fibroblast activity reflect the early properties identified in impaired wound healing, and a dysregulated immune system promotes chronic inflammation with impaired function. Together these changes increase patient susceptibility to barrier breach, impaired healing, and persistent infection. Current clinical trials aiming to address these early cutaneous changes include moisturisers targeting foot xerosis^26–28^ suggesting that improved management with therapies that target these early changes in non-injured diabetic skin may help reduce further complications.

There has been insufficient research on acute wound healing processes in diabetic patients to support clinical differences in healing, with the majority of clinical evidence demonstrating increased infection risk. Considering the lack of epidemiologic literature supporting the role of diabetes impairing acute wound healing, additional significant factors leading to the formation and persistence of wounds developing into DFU should be considered, such as location-based neuropathy and lack of surveillance/management of the wound site. Future clinical studies aiming to elucidate potential disparities between diabetic and non-diabetic acute wounding should consider surgery type, wound area and size, glycaemic control, patient comorbidities, and age, as well as increase their scope to measure additional aspects of healing including scarring and wound re-innervation. It may be challenging to collect and control for such data retrospectively, and large prospective studies of surgical procedures would be important to compare the acute wound healing process between diabetic and non-diabetic patients. Of note, many of the reviewed clinical studies focus on patients with T2D, possibly due to its greater incidence and prevalence as compared with T1D. In addition, epidemiologic studies suggest an increased prevalence of DFU in T2D patients compared to T1D.^194^ The additive influence of common comorbidities of T2D, such as obesity, that also alter cutaneous microenvironment and immunity as risk factors for DFU development, should be considered as well.^195^ Nevertheless, the pathophysiology of glucose intolerance affecting cutaneous properties and neuropathy is universal across both types of diabetes, and related targeted therapies will aid in addressing the multifactorial emergence of diabetic complications.

## Figures and Tables

**TABLE 1:** Summary of epidermal and dermal changes in diabetic skin.

Epidermal and dermal changes in diabetic skin		
Barrier	Stiffened, xerotic epidermis with reduced stratum corneum hydration	5,16,19
Keratinocytes	Increased differentiation, decreased migration and proliferation, with increased ROS	31,32,43
Fibroblasts	Inhibition of proliferation and migration alongside accelerated senescence and increased apoptosis	53,56,57
Extracellular matrix	Fibrosis, accelerated ageing induced by glycation	72,74–77
Microvasculature	Disrupted blood flow due to angiopathy, vascular lumen narrowing, and dilation leading to skin atrophy and susceptibility to microbial dysbiosis	55

**TABLE 2:** Summary of changes in immune cell populations in diabetic skin.

Immune changes in diabetic skin		
Macrophages	Increased expression of pro-inflammatory cytokines and M1 phenotype, and decreased phagocytic capability	53,56,57
Monocytes	Hyperglycaemia-induced resistance to chemotaxis and total increase in circulating monocytes	72,74–77
Mast cells	Increased degranulated cells	55
Neutrophils	Increased release of pro-inflammatory cytokines and NETosis, dysregulated ROS production	80,85

Abbreviation: ROS, reactive oxygen species.

## Data Availability

Data sharing not applicable to this article as no datasets were generated during this study. The figure was created with BioRender.com.

## Abbreviations:

AGE: advanced glycation end products
DFUs: diabetic foot ulcers
DSP: distal sensorimotor polyneuropathy
ECM: extracellular matrix
miRNA: microRNA
MMP: matrix-metalloproteinase
RAGE: AGE receptors
ROS: reactive oxygen species
SC: stratum corneum
TEWL: trans-epidermal water loss
